# Developing a dynamic simulation model to support the nationwide implementation of whole genome sequencing in lung cancer

**DOI:** 10.1186/s12874-022-01571-3

**Published:** 2022-03-27

**Authors:** Michiel van de Ven, Maarten IJzerman, Valesca Retèl, Wim van Harten, Hendrik Koffijberg

**Affiliations:** 1 University of Twente, Department of Health Technology and Services Research, TechMed Centre, Enschede, the Netherlands; 2 University of Melbourne, University of Melbourne Centre for Cancer Research (UMCCR), Parkville, Australia; 3 Peter MacCallum Cancer Centre, Department of Cancer Research, Parkville, Australia; 4 University of Melbourne, Centre for Health Policy, Parkville, Australia; 5 Division of Psychosocial Research and Epidemiology, Netherlands Cancer Institute-Antoni Van Leeuwenhoek Hospital (NKI-AVL), Amsterdam, the Netherlands; 6 Rijnstate General Hospital, Arnhem, the Netherlands

**Keywords:** Implementation, Whole genome sequencing, Dynamic simulation modeling, Diagnostics, Oncology

## Abstract

**Background:**

This study shows how dynamic simulation modeling can be applied in the context of the nationwide implementation of Whole Genome Sequencing (WGS) for non-small cell lung cancer (NSCLC) to inform organizational decisions regarding the use of complex and disruptive health technologies and how these decisions affect their potential value.

**Methods:**

Using the case of the nationwide implementation of WGS into clinical practice in lung cancer in the Dutch healthcare system, we developed a simulation model to show that including service delivery features across the diagnostic pathway can provide essential insight into the affordability and accessibility of care at the systems level. The model was implemented as a hybrid Agent-Based Model and Discrete-Event Simulation model in AnyLogic and included 78 hospital agents, 7 molecular tumor board agents, 1 WGS facility agent, and 5313 patient agents each year in simulation time.

**Results:**

The model included patient and provider heterogeneity, including referral patterns, capacity constraints, and diagnostic workflows. Patient preference and adoption by healthcare professionals were also modeled. The model was used to analyze a scenario in which only academic hospitals have implemented WGS. To prevent delays in the diagnostic pathway, the capacity to sequence at least 1600 biopsies yearly should be present. There is a two-fold increase in mean diagnostic pathway duration between no patients referred or all patients referred for further diagnostics.

**Conclusions:**

The systems model can complement conventional health economic evaluations to investigate how the organization of the workflow can influence the actual use and impact of WGS. Insufficient capacity to provide WGS and referral patterns can substantially impact the duration of the diagnostic pathway and thus should be considered in the implementation of WGS.

**Supplementary Information:**

The online version contains supplementary material available at 10.1186/s12874-022-01571-3.

## Introduction

Whole Genome Sequencing (WGS) is a genomic test that sequences the whole genome with one single test compared to targeted gene panels (TGP) that sequence a subset of genes. While WGS has the benefit of more comprehensive diagnostic information, it is currently more expensive at 2925 euro per patient, whereas the cost of current SoC tests ranges from 70 to 400 euro [1]. Furthermore, even though it has steadily declined over time, the turnaround time of 10 working days for WGS [2], not including clinical interpretation, remains longer than the turnaround time of TGPs. The implementation of WGS into routine clinical practice in oncology is ongoing in several countries worldwide [3, 4]. However, the health economic evidence on the optimal use of WGS as a cancer diagnostic is sparse, as only five initiatives are performing a health technology assessment of WGS with a focus on oncology [2]. 

In addition to demonstrating the clinical and economic value of WGS [5], the actual utilization as part of the diagnostic and treatment episode can impact its affordability and accessibility. For instance, WGS can potentially substitute all DNA-based biomarkers and the optimal position of WGS in the biomarker test strategy needs to be determined. Moreover, the impact on the selection, availability, and start of treatments needs to be addressed. This requires consideration of the required capacity to conduct WGS, curate, and interpret the WGS data. These challenges are not unique to WGS but can also apply to other complex and disruptive health technologies, such as proton therapy [6]. Additionally, short-term inefficiencies may arise during implementation. These inefficiencies can be caused by the overcapacity of the existing technology during the transition phase or due to the gradual implementation of the innovative technology [7].

Cost-effectiveness, cost–benefit, or budget impact analyses typically ignore these additional challenges. These analyses focus on the long-term consequences of specific healthcare interventions and do not typically consider organizational constraints. They implicitly assume that demonstrating the benefits of a new technology will ensure an optimal implementation in clinical practice, perhaps led by the fact that HTA agencies do not generally require evidence on how organizational constraints can affect outcomes. However, this may be an unrealistic assumption when considering complex and disruptive health technologies.

Healthcare delivery systems can be characterized as complex adaptive systems [8]. They contain feedback loops and interaction between different system elements, such as patient-provider interactions. Complex adaptive systems can adapt to changes over time and display nonlinear and delayed behavior. For example, WGS reimbursement increases its budget impact initially through increased use. However, economies of scale could decrease the cost per patient and thus reduce its budget impact in the long term. Additionally, reducing the turnaround time of WGS can lead to an increased adoption rate of physicians and, possibly, to improved benefits for patients. By applying the "big picture" (holistic) principle of systems science [9], we can learn more about the health care delivery system as a whole, compared to evaluating its components in isolation.

Traditional health economic evaluation methods such as decision trees and Markov models are usually not flexible enough to reflect the nonlinear and interdependent properties of the healthcare system. Hence, other methods are required when organizational aspects, as well as care process and technology aspects, need to be reflected. Suitable alternative methods should be able to measure the short and long-term consequences to the system and be flexible enough to reflect complex care pathways [10], often seen in precision medicine.

Dynamic simulation modeling (DSM) has been proposed as a potential approach to reflect the complexity observed in the healthcare system [11]. It consists of three modeling paradigms: System Dynamics (SD), Discrete-Event Simulation (DES), and Agent-Based Modeling (ABM) [10]. SD models relationships between the system elements at an aggregate level using stocks and flows and often contain feedback loops. DES is a process-oriented individual-level modeling approach where entities flow through a process that typically contains delays, resource constraints, and queues. ABM is also an individual-level modeling approach, but its agents are active and may display behavior, unlike in DES. While ABM, DES, and SD are not new, the literature on their application in the context of systems science within health technology assessment is sparse [12]. One article in the healthcare setting combines SD and ABM to assess the value of mobile stroke units [13] while considering the disease and population dynamics, the organization of care and its economics.

This paper aims to demonstrate how DSM can be applied to the nationwide implementation of WGS for non-small cell lung cancer (NSCLC) by conceptualizing and constructing a dynamic simulation model. Technical model details are described in Additional file 1. Moreover, we will illustrate how adjustments in the organization of the diagnostic workflow can provide essential insights into the affordability and accessibility of WGS in the care for cancer patients.

## Case study: Whole Genome Sequencing as a clinical diagnostic in lung cancer

### Background

For many tumor types, choosing the optimal treatment for patients with advanced or metastatic disease depends on the outcomes of biomarker testing. Biomarker testing helps select the optimal treatment and avoid overtreatment with ineffective treatments. The role of biomarkers for treatment selection is especially substantial in lung cancer [14]. Therefore, lung cancer is one of the first tumor types for which WGS will potentially be implemented.

However, it is not clear whether the potential value of WGS outweighs the incremental costs that WGS incurs. Its clinical utility is currently limited to those genes for which a targeted treatment is available. Critics assume that current standard of care (SoC) testing that entails the use of TGP and other tests that test one or a few genes, provide enough information for a clinical diagnosis in most cases. However, proponents hypothesize that WGS adds value in cases where SoC would not have identified a biomarker. Recently, a study concluded that the actionable genome shows limited evolution while under therapeutic pressure, meaning that conducting WGS once is sufficient for most patients [15].

The clinical utility of WGS must be weighed against the incremental costs. WGS requires a significant upfront investment due to the required lab facilities and infrastructure for data storage amongst others. Additionally, WGS has a higher cost per patient. Changes in the organization of care, such as adapting diagnostic workflows to accommodate WGS and putting the required infrastructure in place, will help to realize the potential value of WGS. The need to transform health services underlines the importance of assessing the full, system-wide requirements posed by WGS to support its implementation in routine clinical practice.

### Problem conceptualization

#### Current workflows for biomarker testing in the Netherlands

**Fig.1:**
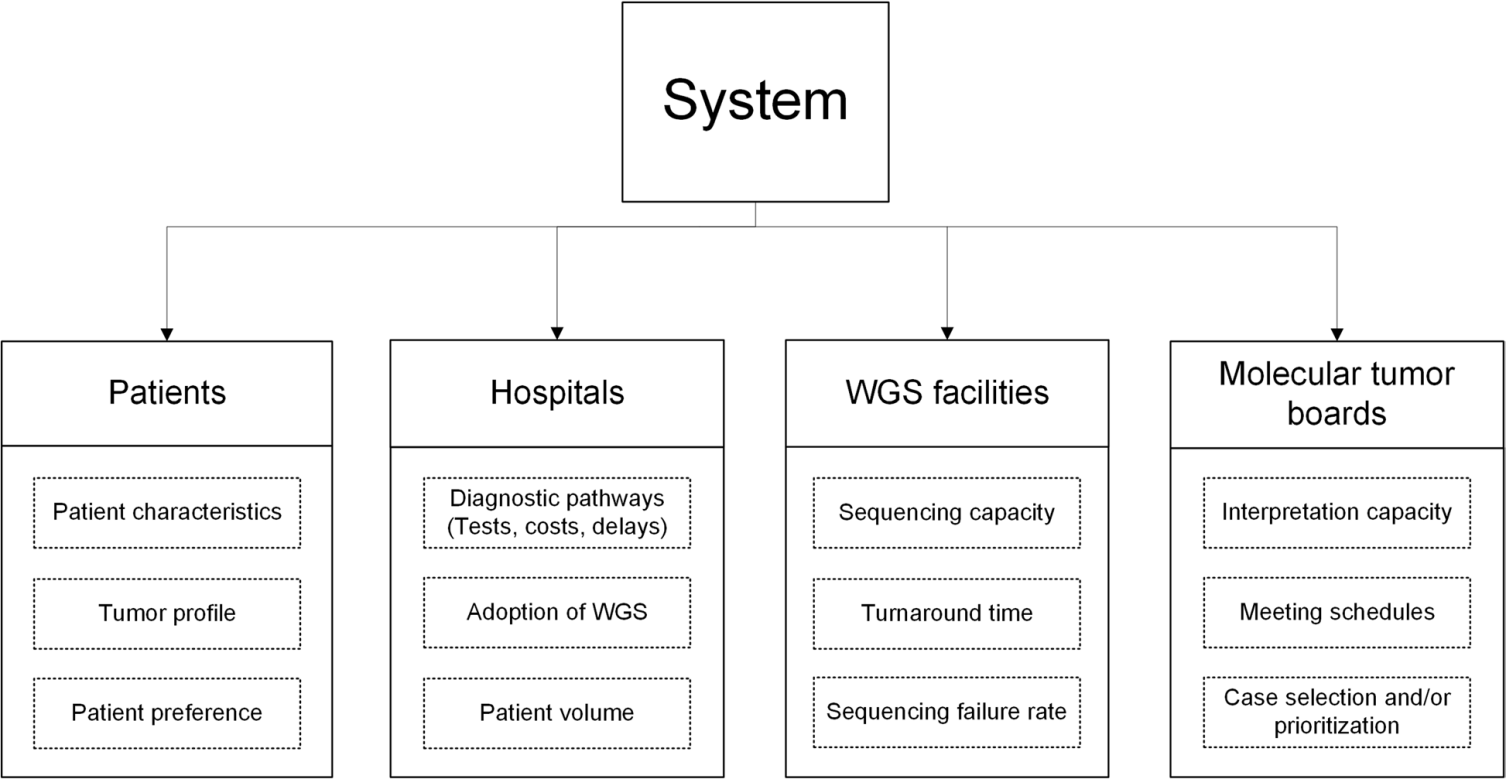
A schematic representation of the healthcare system in which WGS is potentially implemented, comprising the following system elements: patients, hospitals, WGS facilities, and Molecular Tumor Boards. The boxes with dotted lines below each stakeholder represent stakeholder characteristics that may influence the system’s behavior and system outcomes

Figure 1 depicts a schematic representation of the healthcare system considered to implement WGS. The system elements that are shown in Fig. 1 interact with other system elements. For example, patients visit hospitals to be diagnosed and treated, while hospitals use WGS services and molecular tumor boards (MTB) to provide that care. Currently, WGS for cancer patients is primarily used in the clinical research setting as the clinical and/or economic value of WGS has not been demonstrated. One central facility in the Netherlands conducts WGS for cancer patients in hospitals participating in the Centre for Personalized Cancer Treatment study [16]. However, this centralized organization may shift to a regional organization in the near future if hospitals invest in building up their own WGS capacity. Interpretation of the complex genetic information that WGS provides is preferably performed by a group of multidisciplinary experts in an MTB [17]. Currently, the development of MTBs is still in an early phase. Nonetheless, Dutch academic hospitals each organize an MTB who meet regularly.

Biomarker testing for treatment selection is used by all hospitals (*n* = 78) that treat lung cancer patients. Patients can receive treatment with chemotherapy and targeted therapy in most hospitals [18]. Conversely, immunotherapy prescription is concentrated in a subset of hospitals that meet specific quality requirements [19]. Most hospitals that meet these requirements are academic or teaching hospitals or general hospitals with a high patient volume. Enrollment into clinical trials is also initiated via these hospitals. In most cases, patients are referred to one of these hospitals for treatment, or the patient's tumor material is sent to a hospital with a more elaborate testing capability.

#### The potential value of WGS from a systems perspective

##### Diagnostic workflows

A major challenge related to the diagnostic workflows is adapting the current workflows to accommodate WGS. Currently WGS is only used in the clinical research setting in oncology. Therefore, an important step is to determine the patient subgroups that will receive WGS, as the current price level of WGS [1] makes it prohibitive to provide WGS to all patients with lung cancer. This is linked to determining which tests will be substituted by WGS and how tests will be planned. Careful planning is essential, as there is a risk that the time-to-treatment will increase beyond the recommended maxima [18].

Additionally, it has been widely recognized that MTBs should interpret the genetic information that WGS provides [20]. For an adequate interpretation, an MTB should at least consist of clinicians, pathologists, clinical biologists, geneticists, and bioinformaticians [17]. However, there is still a large variety in the composition of MTBs in the Netherlands [21], and MTBs need to be able to cope with a potential increase in the number of patients who receive WGS. This can be achieved, for instance, by automating workflows and setting up clinical decision support systems [22].

The process of conducting WGS differs substantially from the current SoC biomarker tests. When conducting WGS, the patient’s tumor material is sent to a WGS facility. Once sequencing is completed, a report containing the results is sent to the MTB. The MTB discusses the results from WGS and reports an evidence-based treatment recommendation back to the hospital. Ultimately, the treating physicians can, together with the patient, use this recommendation to make a treatment decision. Hence, using WGS involves more and different steps than SoC, which is usually conducted in-house and typically does not use the services of an MTB.

##### Impact of policy decisions

At present, WGS is offered from one location in the Netherlands. While the evidence is still lacking on the effects of centralization [23], focusing all sequencing in one facility can potentially lead to improved efficiency and economies of scale as the throughput increases [24, 25]. However, it is possible or perhaps even desirable that, over time, a regional organization emerges, such that several hospitals can conduct WGS independently. The required capacity to conduct WGS should be carefully predicted, as a decentralized organization potentially leads to overcapacity, similar to what happened with proton therapy in the Netherlands [26]. Overcapacity may be utilized to conduct WGS for new patient indications, for whom a clinical benefit is perhaps not demonstrated yet. This may lead to an increase in the overall budget impact of WGS.

Additionally, the reimbursement status of WGS plays a role in how affordable and accessible WGS is. Presently, WGS is not reimbursed through health insurance in the Netherlands. Especially at the current price level of WGS, the lack of reimbursement presents a substantial barrier to wide-scale use [27]. If the reimbursement status of WGS does not change, only a few hospitals will likely implement WGS into their clinical practice, and then only for narrowly defined patient subgroups. Hence, the reimbursement decision will influence the required sequencing capacity and the likelihood of decentralization.

##### Technical considerations

Technical considerations that separate WGS from other biomarker tests are primarily related to the tissue used for WGS. While WGS is increasingly able to handle formalin-fixed, paraffin-embedded tissue [28], WGS using fresh frozen tissue remains more accurate. Fresh frozen biopsies are not routinely taken, which means that an additional biopsy needs to be taken for WGS. Moreover, biopsies for WGS need to comprise at least 20% of tumor cells for successful sequencing, meaning that approximately 28% of biopsies are not suitable for WGS [29]. These biopsy requirements pose substantial hurdles for successfully conducting WGS, as tumor material is often limited and difficult to access.

### Model implementation

The conceptual model has been implemented as a hybrid dynamic simulation model using both DES and ABM. The SIMULATE checklist [30] was used to describe the systems model and can be found in supplementary file 2. We have opted for a hybrid model as it allows us to benefit from the comparative advantage of each modeling paradigm. Furthermore, both DES and ABM are individual-level modeling paradigms. Individual-level models can make optimal use of available patient-level data to make future events or trajectories dependent on each individual's history and characteristics, which is very informative in the context of precision medicine. For instance, when modeling care pathways, a treatment decision can be based on the outcome of a diagnostic test and patient characteristics.

The model has been developed in AnyLogic 8.3.3 (The AnyLogic Company). AnyLogic is one of several software packages in which multiple DSM model types can be combined in a single, hybrid model, thus providing high flexibility to model developers.

### Model structure

**Fig. 2:**
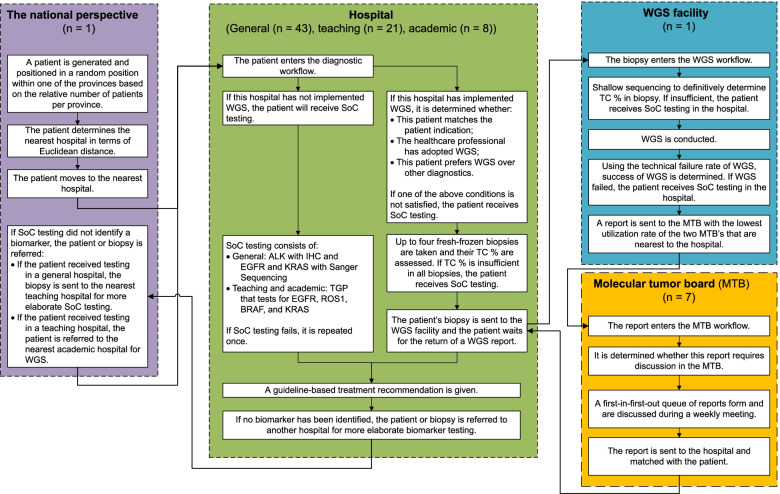
The model structure representing the general flow of patients through the simulation. The model has a multileveled structure: patients, hospitals, the WGS facility, and MTBs are located within the national perspective, which represents the Netherlands. TC %: tumor cell percentage

Figure 2 provides a high-level representation of the model structure. Defining a model boundary is a necessary but subjective decision. The focus of this study is on the required changes in the organization of care. Therefore, system elements that have the largest potential influence on how care is organized or system elements most affected by changes in the organization of care are included in the model.

A hypothetical stage IV NSCLC patient who requires biomarker testing for the initial diagnosis is generated. Upon entering the nearest hospital's workflow, it is determined whether that specific hospital has implemented WGS and whether the patient matches the indication. If the patient receives SoC, all care processes are conducted within that hospital. If the patient should receive WGS, it is assessed whether the pathologist has adopted WGS and whether the patient prefers WGS over SoC. Subsequently, the patient's biopsy is sent to the WGS facility (*n* = 1), and once sequencing is completed, a report is sent to the MTB (*n* = 7). Finally, once either SoC or WGS has been concluded, a guideline-based treatment recommendation is given. Thus, the model's starting point is the diagnosis of stage IV NSCLC, and the endpoint is either death during the diagnostic pathway or the conclusion of the diagnostic pathway.

All hospitals that provide biomarker testing for lung cancer patients are reflected in the model. Hospitals are stratified according to type: general (*n* = 43), teaching (*n* = 21), and academic hospitals (*n* = 8). They differ in the testing strategy they employ. General hospitals have a relatively simple testing strategy; they test ALK rearrangement status using IHC and test the EGFR and KRAS oncogenes' mutation status with Sanger Sequencing. Teaching and academic hospitals test PD-L1 expression and ALK with immunohistochemistry (IHC) and use the same TGP to test for EGFR, ROS1, BRAF, and KRAS. It is assumed that these tests are conducted in parallel.

If SoC testing in a general hospital did not identify a biomarker, that patient is referred to a teaching hospital. If the biomarker testing strategy in a teaching hospital also did not identify a biomarker, that patient is referred to an academic hospital. Academic hospitals have implemented WGS for referred patients and patients for whom SoC testing in that academic hospital did not identify a biomarker. If biomarker testing in the academic hospital also did not identify a biomarker, that patient is not referred further. Hence, WGS is implemented as a last-resort diagnostic test. A technical model description, describing the different agent types and parametrization is available in Additional file 1.

### Model transparency and validation

We aimed to create model transparency by providing a clear description of the model and its software implementation. Furthermore, the model has been uploaded to AnyLogic Cloud [31]. Systems models are typically relatively complex and, therefore, difficult to extensively validate. In this case, validating the outcomes of a scenario in which WGS is not used against real-world data was not possible as those data were not available. Achieving face validity is often seen as an important first step [32]. Face validity was achieved through several discussions with stakeholders during and after model development to discuss modeling choices, assumptions, and outcomes. During model development, interactive discussions were held with the Technology Assessment of Next Generation Sequencing in Personalized Oncology (TANGO) consortium [33], which investigates the added value of WGS for clinical diagnostics in the Netherlands. This group consisted of experts on oncology, pathology, genetics, bioinformatics, ethics, and health economics. Once model development was concluded, an interactive discussion with patient representatives, stakeholders from the current genomic services provider, and the TANGO consortium was organized to evaluate whether the model’s face validity was sufficient.

### Model-based analysis

Sensitivity analyses were conducted for model verification and to illustrate several relationships within the model. The following parameters were varied: the cost of WGS, the percentage of patients who need to be referred to another hospital that are referred, and the capacity to conduct WGS. For each parameter setting, the model was run 500 times to quantify the stochastic uncertainty in the outcomes [34]. To achieve stable outcomes, each simulation ran for 2000 days. With an annual expected patient population of 5313 [18], each run approximately simulated 29,000 patients.

### Results

#### The cost of WGS

**Fig. 3:**
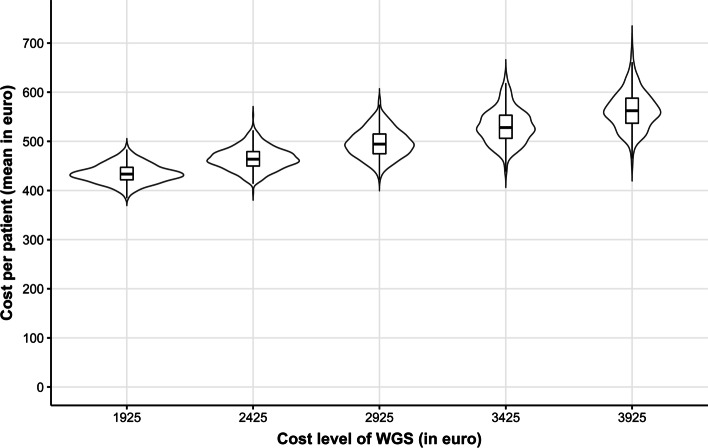
The impact of the cost of WGS on the mean cost per patient across all patients. The length of each violin symbolizes the uncertainty in the estimate of the mean cost per patient. The boxplots show the median and interquartile ranges. The horizontal axis represents the current cost level of WGS (2925 euro) [1] and hypothetical cost levels with 500 euro increments

Figure 3 shows the impact of changes in the cost of WGS on the mean cost per patient. Figure 3 includes all patients; patients who received only SoC and patients who received both SoC and WGS. The changes in the cost of WGS have no impact on the mean cost of patients who did not receive WGS and only received SoC. Additionally, not every patient received WGS, and therefore, the mean cost per patient does not increase on a one-to-one basis with the cost level of WGS.

#### Referral patterns for biomarker testing

**Fig. 4:**
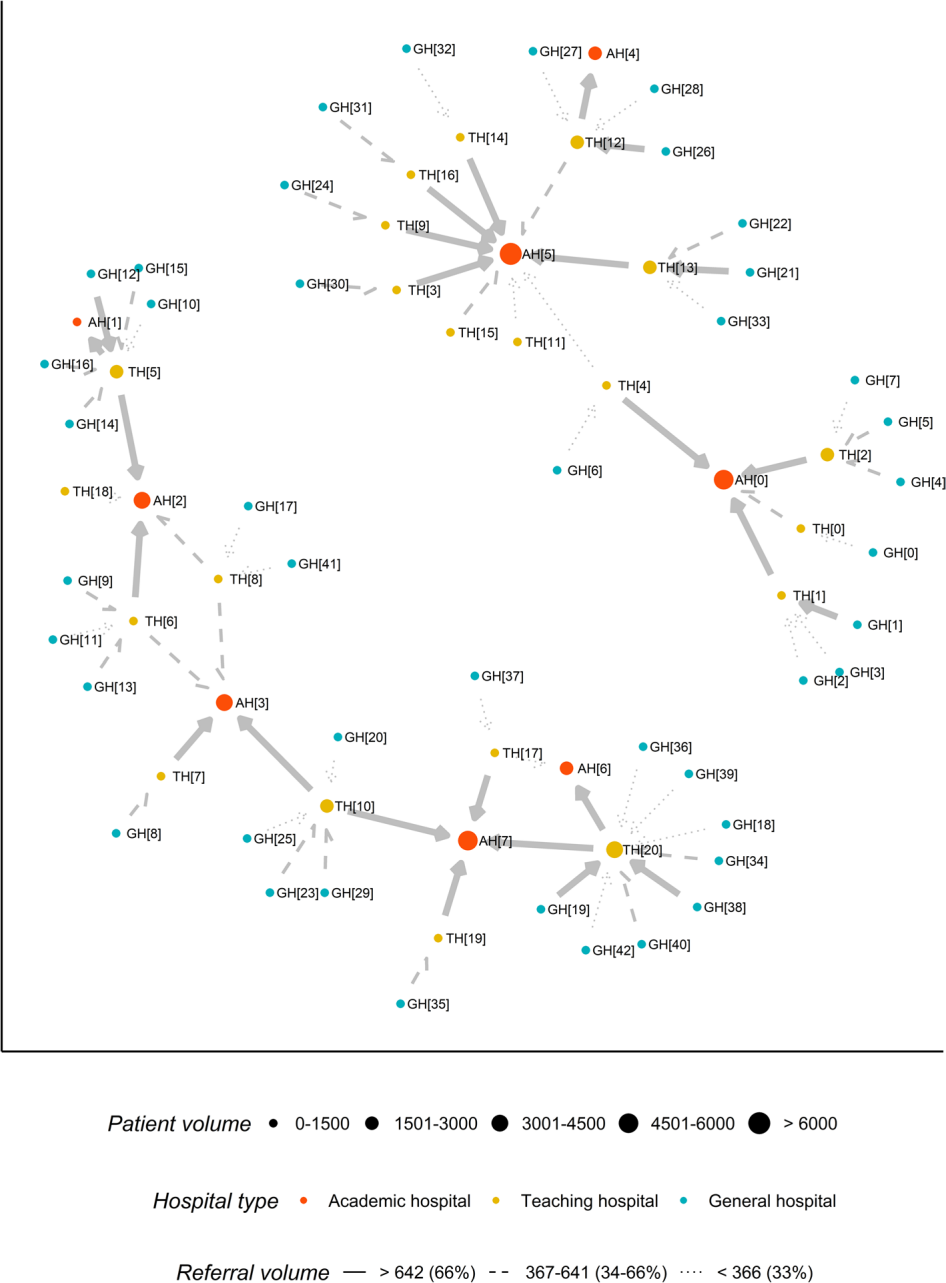
Hospital networks in one simulation run. The nodes represent hospitals. Node size represents the total patient volume in the simulation run. Node color represents the hospital type. The edge line type and edge width represent the referral volume expressed in the number of patients between two hospitals. The space between hospitals does not represent geographic distance

All hospitals are placed in networks with other hospitals to facilitate referrals of patients among hospitals. To benefit from more extensive biomarker testing, general hospitals refer patients to the nearest teaching hospital, and teaching hospitals refer patients to the nearest academic hospital. Patients who received WGS will not be referred, as there is no additional biomarker test available. Figure 4 shows these hospital networks, as well as the size of the referral flows and patient volume per hospital. For example, general hospital 5 (GH[5]) has a patient volume of below 1500 patients and referred between 367 and 671 patients to teaching hospital 2 (TH[2]). TH[2] has a patient volume of between 1501 and 3000 patients. While TH[2] also received referred patients from general hospitals 4 and 7 but refers only to academic hospital 0 (AH[0]), with a referral volume exceeding 642 patients. AH[0] has a patient volume of between 4501 and 6000 patients. AH[0] does not refer patients, but did receive referred patients from teaching hospitals 0, 1, 2, and 4. Note that Fig. 4 is a visualization based on data from one simulation run. In each simulation run, the distribution of hospitals across networks can vary, but how patients are referred is constant across runs. From Fig. 4, we can observe that hospitals vary in patient volume, patient referrals (both sending and receiving), and the degree of the relative importance of hospitals in the network.

**Fig. 5:**
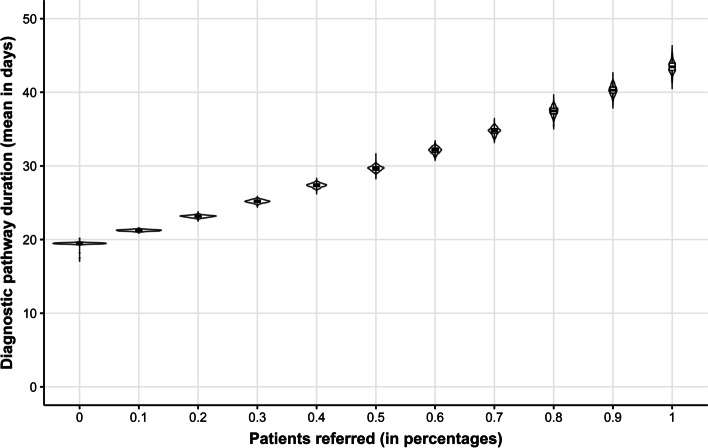
The impact of the percentage of patients who should be referred to a different hospital on the diagnostic pathway duration is expressed in days. The assumption underlying referrals is that all patients for whom no biomarker was identified in their current hospital are patients who should be referred if there is more elaborate biomarker testing available elsewhere. The length of each violin symbolizes the uncertainty in the estimate of the mean diagnostic pathway duration. The boxplots show the median and interquartile ranges

Patients are referred to other hospitals if no actionable target has been found and more elaborate biomarker testing is available elsewhere. Figure 5 shows that a higher percentage of referrals lead to, on average, a longer diagnostic pathway. The diagnostic pathway's mean duration increases when more patients are referred due to a model mechanism that extends the diagnostic pathway for several days when a patient is referred, reflecting that referrals cause a delay [18]. Moreover, the uncertainty in the mean diagnostic pathway duration increases once more patients are referred.

#### Capacity constraints for WGS

**Fig. 6:**
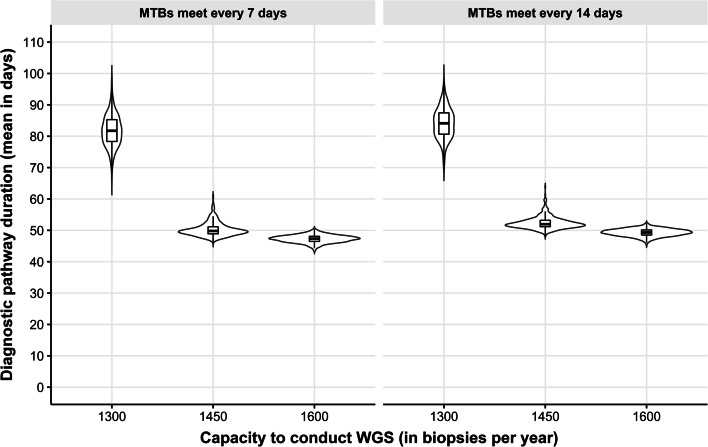
The impact of capacity constraints to provide WGS on the diagnostic pathway duration expressed in days for patients who received WGS. The length of each violin symbolizes the uncertainty in the estimate of the mean diagnostic pathway duration expressed in days. The boxplots show the median and interquartile ranges

Figure 6 illustrates how constraining the capacity to conduct WGS and the MTB capacity to give a clinical interpretation of the WGS report impacts the percentage of patients who died before receiving a treatment recommendation. Figure 6 is stratified by MTB meeting frequency; weekly or every two weeks. Once the sequencing capacity is below 1600 biopsies annually, which is enough capacity to prevent long queues in this scenario, the diagnostic pathway's mean duration increases. At a capacity of 1450 biopsies annually, the effects are noticeable but not as extreme compared with a capacity of 1300 biopsies annually. This extreme undercapacity leads to a significantly increased mean duration of the diagnostic pathway and increased uncertainty surrounding that mean estimate. The MTB meeting frequency is also a form of capacity constraint, as it affects the waiting time for the clinical interpretation of WGS results. If MTBs meet once every 14 days, the duration of the diagnostic pathway increases slightly, approximately equal to seven days.

## Discussion

In this study, we have demonstrated how DSM can be utilized to develop a systems model that can be used to evaluate the impact of the nationwide implementation of WGS for NSCLC. The study first described the intended use of WGS in oncology and the challenges related to the organization it faces. Subsequently, this real-world problem has been translated into a proof-of-concept dynamic simulation model reflecting the heterogeneity in patients and providers, behavioral aspects, and geographic variation. Visualization of hospital networks and the sensitivity analyses have illustrated that aspects related to the organization of care, such as capacity constraints and referral patterns, can substantially impact outcomes of interest such as the duration of the diagnostic pathway and the cost per patient.

The main benefit of a DSM with a system-level perspective is the ability to reflect care processes, geographic variation, and behavioral aspects, such as patient preferences and the adoption by individual physicians, which are typically neglected in traditional (Markov type) simulation models [35].

Moreover, dynamic simulation models can reflect the organization of care across multiple heterogeneous providers. Therefore, a dynamic simulation model can investigate multiple different domains that may influence a health technology's actual use and outcomes in a particular context. Oncology and genomics are fast-moving fields. To make the model more futureproof, the model is set up in a way that potential future developments can be reflected in the model relatively straightforwardly and would not impose changes to the model structure.

For instance, in the systems model, it is not necessary to assume that the implementation of WGS is immediate and perfect. It is more plausible that the implementation of WGS will be gradual and that the organization of WGS will affect the benefits of WGS and vice versa, which can be appropriately reflected in a systems model. Figures 3, 5, and 6 show that a traditional model assuming perfect and complete implementation of WGS and assuming unlimited capacity would produce different outcomes regarding the diagnostic pathway’s duration and mean cost per patient.

The systems model in this paper combines mechanisms from ABM and DES. A hybrid model benefits from the comparative advantage of each modeling paradigm, allowing the efficient simulation of processes, events, and resources, as well as behavior and interactions. This combination would be much harder to achieve and requires more assumptions when using either DES or ABM by itself. A practical benefit of creating a hybrid model is that it offers flexibility to the model developer, which is valuable if unforeseen model components need to be included. Note that no transformation of inputs and outputs is necessary as both ABM and DES are individual-level modeling paradigms, making it straightforward to combine them.

Given the increasing complexity of the healthcare system, systems models that focus on the organization of care may become more desirable in the future. Though, a systems model requires different and additional data to reflect the system's interdependencies, such as referral patterns, and provider heterogeneity, such as the SoC testing strategy in hospitals. Moreover, conceptualizing the problem and defining model boundaries with stakeholders requires a larger time investment than traditional health economic models. Therefore, it would be worthwhile to determine beforehand whether a systems model would provide additional insights compared to a traditional cost-effectiveness model or budget impact analysis. Whether there are benefits depends on the characteristics of the health technology in question, the anticipated required changes to the organization of care for optimal implementation, the diversity of involved stakeholders, and the disruptive nature of the health intervention.

A fundamental challenge for all modelers is defining the model structure required to represent the real-world problem adequately. As our model aims to inform organizational decisions regarding the use of WGS, we naturally focused on the flow of patients and information between the involved actors. To achieve face validity, we have used multiple interactive discussions with stakeholders, before and during model development, to ensure our model was fit for purpose and credible. To minimize model complexity [36], we added model components incrementally when warranted by the stakeholder discussions. Nonetheless, it is possible that involving different stakeholders might have led to slightly different modeling decisions.

Another challenge is the degree of detail that is reflected in the model. That decision was partly driven by data availability. Assumptions were made if the data were lacking for model components that were deemed critical. For instance, we assumed that SoC testing was identical in hospitals of the same type. Therefore, it may not be a perfect representation of the actual test strategy in all hospitals. However, we have aimed to match the degree of detail reflected in the model with the type of research question this model will answer. The model we developed will be used for tactical and strategic purposes. Therefore, details that probably do not impact the outcomes significantly can be omitted. Omitting unnecessary details leads to a less complex model, which reduces the model's computational burden and makes it easier to validate the model with stakeholders.

Many aspects of a systems model will, by design, be country-specific. Hence, generalizability may be limited, depending on the extent to which the organization of care differs across countries. Nevertheless, the basic concepts of developing and implementing a systems model are independent of a country-specific context and can be applied generally.

There are many exciting avenues for future research. Given that the healthcare system comprises intelligent agents that can adapt to new circumstances [30], it would be challenging and exciting to explore the healthcare system's adaptive and dynamic behavior and incorporate it into the systems model using different implementation scenarios to WGS. Additionally, developing creative ways to validate the model structure, such as by comparing the consequences of natural experiments in the healthcare system with model outcomes, would be valuable.

## Conclusions

In this study, we have demonstrated how DSM can be applied to the nationwide implementation of WGS for NSCLC. Sensitivity analyses have illustrated that aspects related to the organization, such as capacity constraints and referral patterns, can substantially impact outcomes. The systems model can complement conventional health economic evaluations to investigate how aspects in organizational and behavioral domains influence the actual use and impact of WGS.

## Supplementary Information

**Additional file 1:** **Appendix 1.** Model description

**Additional file 2.** The SIMULATE checklist

## Data Availability

The datasets analyzed during the current study are available from the corresponding author on reasonable request.
