# Supplementary material for: Developing a dynamic simulation model to support the nationwide implementation of whole genome sequencing in lung cancer
Source: BMC Med Res Methodol. 2022 Mar 27;22:83. doi: 10.1186/s12874-022-01571-3 (PMC8962015; doi:10.1186/s12874-022-01571-3)
Supplement: Supplementary file 1 — Additional file 1: Appendix 1. Model description [file 12874_2022_1571_MOESM1_ESM.docx]

# Appendix 1: Model description

The purpose of this model is to show how dynamic simulation modeling can be applied in the context of the nationwide implementation of WGS for NSCLC to inform organizational decisions regarding the use of complex disruptive health technologies and how these decisions affect their potential value. This document is intended to provide more detailed information on the model implementation in AnyLogic. The model has been uploaded to AnyLogic Cloud and is publicly accessible (1).

The model is initially populated with six different agent types: patient, general hospital, teaching hospital, academic hospital, WGS facility, MTB. These agents are all placed within a top-level agent (Main) that represents the Netherlands. A distinction is made between three hospital types to reflect differences in diagnostic testing capabilities. During model runtime, agents of a seventh type, WGS biopsy, are generated within in academic hospital-agents.

## Agent types

Table 3 lists the model input parameters including a reference for each parameter.

### Main

The top-level agent in a hierarchical model represents the highest level of abstraction and serves as the stage for the other agents. In this model, the top-level agent represents the Netherlands, shown in figure 1.
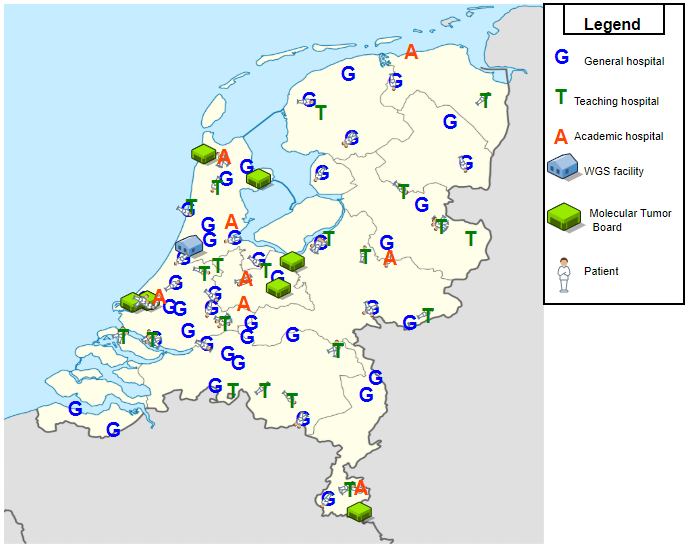
 Figure 1. Top-level agent or highest abstraction level in the model representing the Netherlands. Map of the Netherlands was retrieved from Wikimedia Commons: https://commons.wikimedia.org/wiki/File:Netherlands_location_map.svg

Since AnyLogic does not provide a method to reflect geographic variation in agent populations, we have implemented our own approach to reflect geographic variation in terms of patient population size. This is implemented as follows. The relative number of patients per province (2) is calculated. This relative number is then used to slice a range from 0 to 1 into 12 segments, one for each province. The size of each segment is based on the relative number of patients per province, which is displayed in table 1. Subsequently, a random number from a uniform distribution between 0 and 1 is drawn. It is then checked in which segment this random number falls. The province to which this segment corresponds is selected to place the newly generated Patient-agent.

Table 1. Input data for agent placement

| **Province** | **Patient-agents** |
| --- | --- |
| Groningen | 0.0349 |
| Friesland | 0.0371 |
| Drenthe | 0.0291 |
| Overijssel | 0.0700 |
| Flevoland | 0.0183 |
| Gelderland | 0.1220 |
| Utrecht | 0.0610 |
| Noord-Holland | 0.1514 |
| Zuid-Holland | 0.2090 |
| Zeeland | 0.0250 |
| Noord-Brabant | 0.1585 |
| Limburg | 0.0838 |

Once a province is determined, the patient is placed at a random location in the specified province. Thus, this approach only considers differences between provinces, and not within provinces. In contrast, a specific number of general hospitals, teaching hospital, academic hospital, WGS facility, and MTB agents is placed within each province. In total, 43 general hospitals, 21 teaching hospitals, and 8 academic hospitals (including a comprehensive cancer center) are placed in Main which represents the national perspective and contains all other agents.

#### Treatment algorithm

Figure 2 shows the algorithm that is used in all hospitals to provide a guideline-based treatment recommendation once the diagnostic workflow has been concluded, and is based on the NCCN clinical practice guidelines (3). It uses the outcomes of the biomarker tests for each patient. In cases where the patient-agent is eligible for both immunotherapy and targeted therapy, the latter takes precedence over the former.


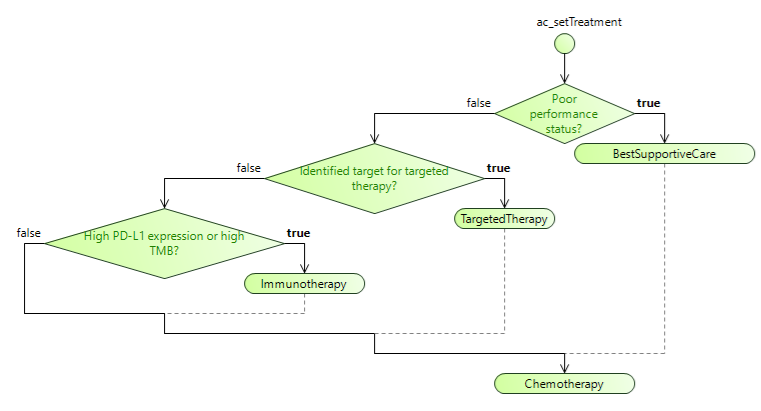


Figure 2. The treatment algorithm used to give treatment recommendations.

### Patient

This agent type reflects patients who have been diagnosed with stage IV NSCLC, but who have not yet received biomarker testing or treatment. The patient population is open, meaning that each time period new patients are being generated. The number and interarrival time of patients is determined using a Poisson distribution (λ = 5313). When a patient-agent is generated, the patient-agent selects the nearest hospital, irrespective of hospital type, and moves to that hospital to receive biomarker testing. Patient-agents will only be removed from the model once their diagnostic pathway has been concluded or because they have died.

### General, teaching, and academic hospitals

All three types of hospital-agents contain a DES workflow that reflects the diagnostic pathway for patients with stage IV NSCLC at a high level, which is depicted in figure 3 and 4. Either a patient receives standard of care (SoC) biomarker testing or WGS. In the model, only academic hospitals have implemented WGS.

When a patient-agent enters the workflow in one of the hospitals, it is checked whether this specific hospital has implemented WGS. If that is the case, it is checked whether the patient matches the indication for WGS. If the patient does not match the indication for WGS, the patient will receive standard of care biomarker testing. If the patient matches the indication for WGS, it is further checked whether the patient prefers WGS over SoC (P = 0.90) and whether the physician has adopted WGS (P = 0.90). Both conditions need to be true for the patient to receive WGS.

Subsequently, a new agent is generated, representing a biopsy to be used for WGS. To be suitable for WGS the biopsy must contain at least 20% tumor cells. In reality, this is visually inspected by a pathologist. In the model, this is evaluated within the hospital care flow. The probability that a given biopsy contains at least 20% tumor cells is determined using a beta distribution (p = 570, q = 297, min = 0, max = 1). If the biopsy indeed contains at least 20% tumor cells, the biopsy is sent to most nearby WGS facility-agent, where it enters a workflow that represents the process of conducting WGS.


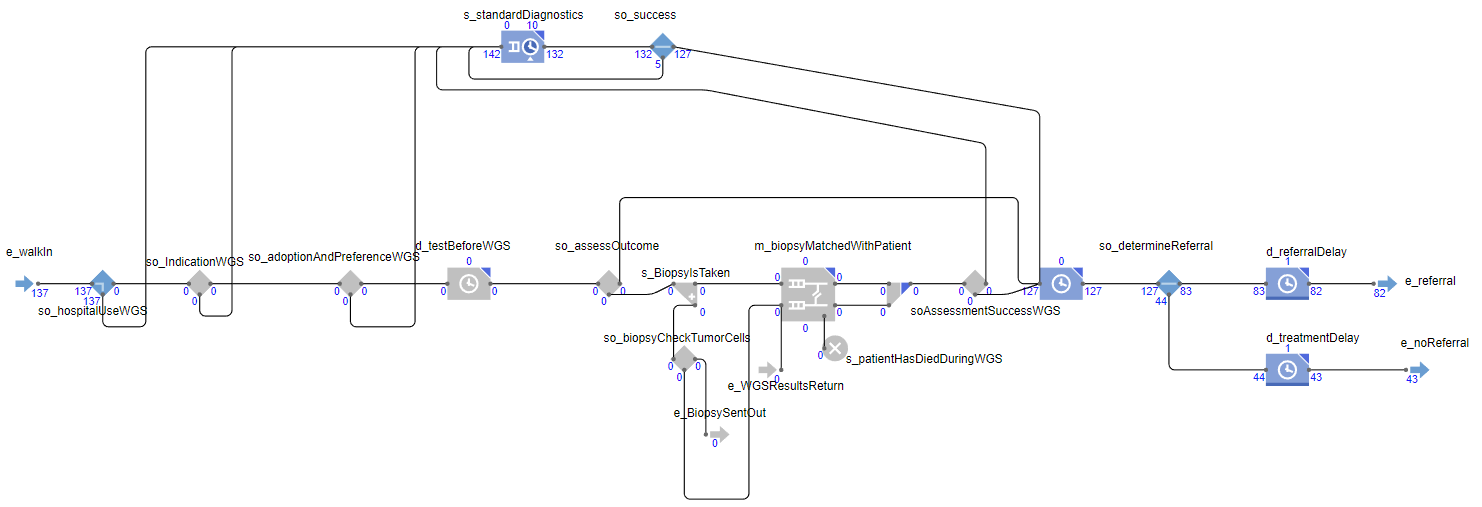


Figure 3. Care workflow in general and teaching hospitals. The top pathway is for SoC biomarker testing, while the bottom pathway is for WGS, which is not in use (greyed out) in general and teaching hospitals. The numbers below each block represent that number of patients that are currently in that block or that have passed that block.

The patient-agent remains located in the hospital and waits while WGS is being conducted. Once the biopsy returns from the WGS facility, it is checked whether WGS was conducted successfully. This is dependent on whether the biopsy passed quality control check in the WGS facility and the technical success rate of WGS, but this is further discussed in section 1.1.4. If it was unsuccessful, the patient-agent will receive SoC. If WGS was successful and the MTB has interpreted the resulting genetic information, the biopsy-agent is sent back to the hospital-agent where it links up with its original patient-agent.

If the patient receives SoC biomarker testing, all processes take place within the hospital. Both SoC and WGS incur a delay. Once either SoC or WGS has been completed, a guideline-based treatment decision is made based on the test outcomes and the performance status of the patient.

#### Biomarker testing strategies

There is a difference in the SoC biomarker testing strategy among hospital types. General hospitals have the simplest test strategy, as they test genes PD-L1 and ALK with immunohistochemistry (IHC), and EGFR and KRAS using Sanger Sequencing. It is assumed that these tests are conducted in parallel. Teaching hospitals test for ALK and PD-L1 using IHC in parallel with a targeted gene panel that tests for KRAS, BRAF, EGFR, and ROS1. Academic hospitals use the same SoC test strategy as teaching hospitals for non-referred patients. Additionally, they conduct WGS for referred patients and for non-referred patients for whom the combination of the targeted gene panel and IHC test did not result in the identification of a biomarker. For simplicity, we have assumed that the biomarker test strategies are identical for all hospitals of each hospital type.

If a patient is KRAS mutation-positive, that patient cannot be EGFR mutation-positive. All other mutations are not considered mutually exclusive in the model. The model reflects that biomarker testing will occasionally fail. When WGS fails, the patient shall receive WGS. If SoC testing fails, SoC testing will be repeated once.

#### Diagnostic turnaround times and delays

The turnaround times of diagnostics and the length of the treatment delay in the care workflow of hospitals are obtained from a survey that was distributed in 2019 among 17 oncologists employed in different hospitals and hospital types. Oncologists were asked to map the timeline of individual steps in the care pathway until treatment initiation. Table 2 lists the survey data for each oncologist. Discrete empirical distributions were created for each delay type (result molecular diagnostics, result PD-L1 test, and treatment delay) and for each hospital type. The values for the empirical distributions for treatment delay were determined by subtracting either the delay for result molecular diagnostics or result PD-L1 test, whichever one is largest, from the time until treatment initiation. The resulting number is the time between conclusion of the diagnostic pathway and treatment initiation, thus, the treatment delay.

Given our assumption that all SoC tests are conducted in parallel, to determine the delay for SoC, a random value was drawn from the empirical distributions for result molecular diagnostics and result PD-L1 test for the matching hospital type. The largest value of the two was used as the delay for SoC.

Table 2. Survey data for diagnostic turnaround times and treatment initiation

| Oncologist | Hospital type | Request molecular diagnostics or PD-L1 test (start interval) | Result PD-L1 test | Result molecular diagnostics | Treatment initiation |
| --- | --- | --- | --- | --- | --- |
| 1 | Academic | 0 | 15 | 21 | 28 |
| 2 | Academic | 0 | 4 | 10 | 12 |
| 3 | Academic | 0 | 14 | 14 | 18 |
| 4 | Academic | 0 | 10 | 21 | 21 |
| 5 | Academic | 0 | 9 | 21 | 28 |
| 6 | Academic | 0 | 15 | 20 | 30 |
| 7 | General | 0 | 4 | 21 | 22 |
| 8 | General | 0 | 10 | 10 | 14 |
| 9 | General | 0 | 7 | 14 | 19 |
| 10 | Teaching | 0 | 5 | 10 | 14 |
| 11 | Teaching | 0 | 8 | 14 | 20 |
| 12 | Teaching | 0 | 12 | 14 | 21 |
| 13 | Teaching | 0 | 14 | 21 | 25 |
| 14 | Teaching | 0 | 15 | 23 | 32 |
| 15 | Teaching | 0 | 12 | 15 | 20 |
| 16 | Teaching | 0 | 6 | 12 | 20 |
| 17 | Teaching | 0 | 7 | 10 | 14 |

#### Hospital networks and referrals

At the start of each simulation, nearby hospitals form a network. More specifically, general hospitals connect to the nearest teaching and nearest academic hospital, and teaching hospitals connect to the nearest academic hospital. These hospital networks are used to facilitate referrals between hospitals. If biomarker testing in a general hospital has not identified a biomarker, that patient will be referred to the teaching hospital that is connected to the general hospital in the network. Similarly, if biomarker testing in a teaching hospital has not identified a biomarker, that patient will be referred to the academic hospital in the network. Academic hospitals do not refer patients elsewhere, as they have most elaborate testing capabilities.


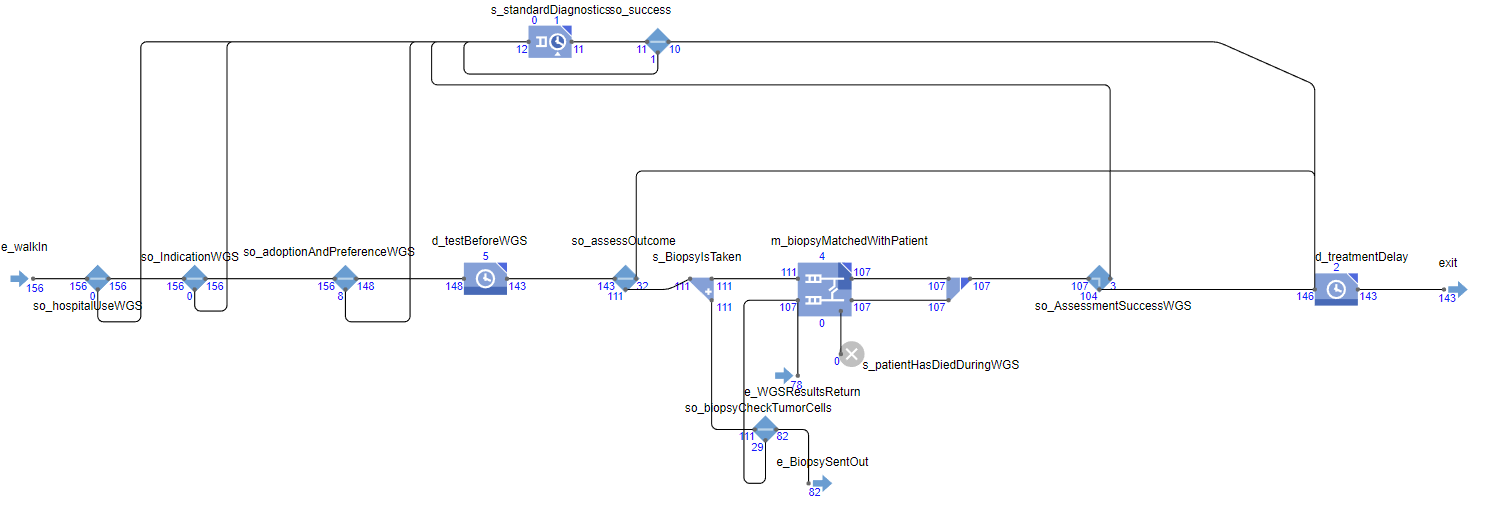


Figure 4. Care workflow in academic hospitals. Both the trajectory for SoC biomarker testing (top pathway), and the trajectory for WGS (bottom pathway) are active. The numbers below each block represent that number of patients that are currently in that block or that have passed that block.

### WGS facility

This agent-type is responsible for conducting WGS. Even though the tumor cell percentage is visually inspected by a pathologist in the hospital, a definitive check, using shallow WGS, is performed in the WGS facility. The probability that a given biopsy passes this definitive check is determined using a beta distribution (p = 570, q = 28, min = 0, max = 1). If it is confirmed that the biopsy contains enough tumor cells, WGS will be conducted. Otherwise, the patient will receive SoC biomarker testing. The workflow presented in figure 5 reflects this workflow. When WGS is concluded, the biopsy-agent is sent to the MTB-agent that is closest to the hospital-agent the biopsy-agent was sent from.


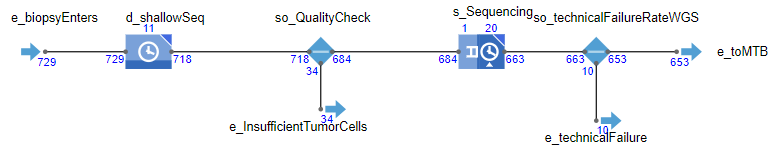


Figure 5. WGS facility workflow. The numbers below each block represent that number of biopsies that are currently in that block or that have passed that block.

From figure 5, we can observe that both the quality check and sequencing itself incur a delay. For the entire sequencing workflow delay, we draw for each biopsy-agent one value from a truncated normal distribution (min = 7, max = 21, mean = 14, sigma = 5). The delay incurred by the quality assessment accounts for 25% of this delay, and the sequencing itself accounts for the remaining 75%. These percentages are based on assumptions. This is implemented as such, given that only the total turnaround time of WGS is known and the turnaround time of individual components of this process is unknown. Even though the 25%/75% split is an assumption, the mean turnaround time of WGS is based on literature.

In the base-case analysis, we assumed that the capacity to conduct WGS is unlimited. While it is unlikely that the capacity is truly unlimited, it is plausible that the capacity is sufficiently large to have little or no impact on outcomes. The capacity to conduct WGS was varied in the sensitivity analyses to illustrate the potential effects of these constraints on outcomes.

### MTB

MTB-agents contain a workflow the represents at a high level the workflow used for interpreting the genetic information from WGS. The fact that not the interpretation itself but rather the infrequent meeting schedule of the MTB causes a delay in the diagnostic pathway is reflected in the modeled workflow. Figure 6 illustrates the workflow within the MTB-agents. First, it is determined if the complexity of this case warrants discussion in the MTB. For simplicity, we have assumed that all patients that have received WGS need to be discussed in an MTB. Subsequently, a first-in-first-out (FIFO) queue of biopsy-agents that require interpretation is formed. However, interpretation requires experts, and they meet according to a given schedule. Thus, if a biopsy-agent narrowly missed a meeting, it waits in the queue until the next meeting is scheduled. When interpretation has concluded, the biopsy-agent is sent back to the original hospital where it is linked to the patient it was taken from.

To which MTB the report is sent to from the WGS facility, is determined based on the distance to the hospital. This is either the nearest MTB to the hospital, or the second nearest MTB if the nearest MTB has a utilization rate greater than the utilization rate of the second nearest MTB.


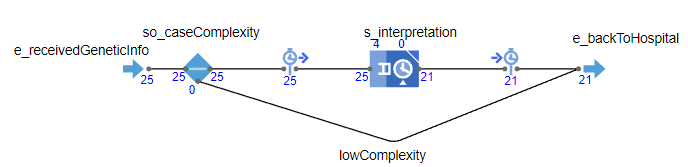


Figure 6. MTB workflow. The numbers below each block represent that number of patients that are currently in that block or that have passed that block.

### WGS biopsy

An agent of this agent-type is only generated whenever a patient will receive WGS. Once WGS has concluded, the biopsy-agent and the patient it originates from are matched using a shared unique identifier and merged into one agent. Figure 7 shows the statechart that controls the movement of biopsy-agents between hospital-agents, WGS facility-agents, and MTB-agents. Transitions between states are fired using messages whenever the biopsy-agent has received a specific point in the workflow in the WGS facility-agent or the MTB-agent. For example, once WGS is concluded, the workflow within the WGS facility sends a message (“Move to MTB”) to the biopsy-agent, which fires a transition in the statechart, and the biopsy-agent then moves to the MTB.


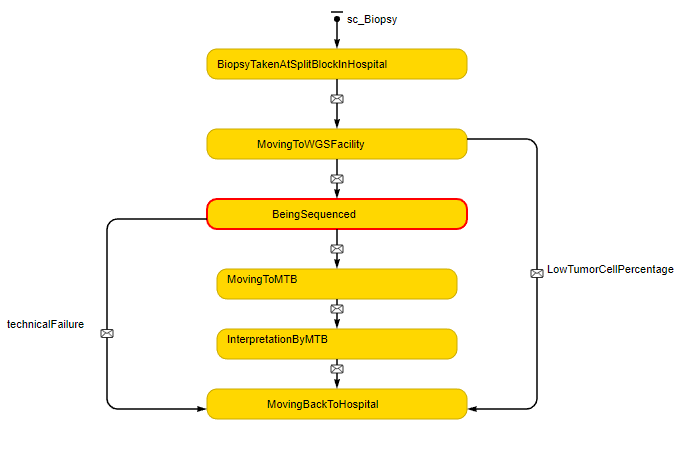


Figure 7. Biopsy-agent statechart

## Model parameterization

Table 1 lists the model input parameters, which are based on literature, expert opinion, or assumptions. The model is intended as a proof-of-concept and an illustration of how dynamic simulation modeling and systems science can be utilized in HTA to inform organizational decisions regarding the use of complex disruptive health technologies. That goal can be achieved even if, for some parameters, data is lacking, and assumptions had to be made.

Table 3: Model input parameters

| **Parameter category** | **Parameter name** | **Description** | **Value** | **Reference** |
| --- | --- | --- | --- | --- |
| **Global** | Annual patient rate | Number and interarrival time of patients diagnosed with stage IV NSCLC each year | Poisson(λ = 5313) (integer) | (4). The type of distribution for the interarrival time is based on assumption. |
|  | MTB count | MTB agent population size | 8 (integer) | (5) |
|  | WGS facility count | WGS agent population size | 1 (integer) | Reflects the current situation in the Netherlands |
|  | Patient indication WGS | Probability of 0 or 1 that a given patient matches the patient indication for whom WGS is indicated | $P\left( X \right\vert$  $referred to academic$  $hospital=1) \vert$  $P\left( X \right\vert SoC in academic$  $hospital identified$  $biomarker=0) = 1$  $P\left( X \right\vert referred to academic$  $hospital=0) \vert P\left( X \right\vert$  $SoC in academic$  $hospital identified$  $biomarker=1) = 0$  (fraction) | Assumption |
|  | WGS Adoption rate | The proportion of physicians who have adopted WGS | 0.90 (fraction) | Assumption |
|  | Patient preferences | Probability that the patient-agent prefers WGS over SoC | 0.90 (fraction) | Assumption |
|  | Proportion of patients who are discussed MTB | The proportion of patients who received WGS that are discussed in an MTB | 1 (fraction) | Assumption |
| **Patient characteristics** | KRAS mutation prevalence | Probability that patient harbors an EGFR mutation | Non-squamous cell carcinoma: Beta(p = 784, q = 1268)  Squamous cell carcinoma: Beta(p = 3, q = 26)  (fraction) | Non-squamous: (6)  Squamous: (7) |
|  | EGFR mutation prevalence | Probability that patient harbors an EGFR mutation. EGFR mutations do not overlap with KRAS mutations. | Non-squamous cell carcinoma: Beta(p = 218, q = 1834)  Squamous cell carcinoma: Beta(p = 1, q = 28)  (fraction) | Non-squamous: (6)  Squamous: (7) |
|  | ALK rearrangement prevalence | Probability that patient harbors an ALK rearrangement | Beta(p = 238, q = 7538) (fraction) | (8) |
|  | ROS1 rearrangement prevalence | Probability that patient harbors a ROS1 rearrangement | Beta(p = 7, q = 530) (fraction) | (9) |
|  | BRAF mutation prevalence | Probability that patient harbors a BRAF mutation | Beta(p = 143, q = 7633) (fraction) | (8) |
|  | MET mutation prevalence | Probability that patient harbors a MET mutation | 0.04 (fraction) | (10) |
|  | RET rearrangement prevalence | Probability that patient harbors a RET rearrangement | Beta(p = 22, q = 975) (fraction) | (11) |
|  | HER2 mutation prevalence | Probability that patient harbors a HER2 mutation | Beta(p = 64, q = 7712) (fraction) | (8) |
|  | NTRK rearrangement prevalence | Probability that patient harbors a NTRK rearrangement | Uniform distribution(0.02, 0.03) (fraction) | (10) |
|  | High PD-L1 expression level prevalence | Probability that patient has a tumor proportion score (TPS) equal or greater than 50% | Beta(p = 181, q = 650) (fraction) | (8) |
|  | Performance status | Patients’ clinical condition on the Eastern Cooperative  Oncology Group Performance Status (ECOG PS) scale | PS 0-1: 0.695  PS 2: 0.192  PS 3: 0.079  PS 4: 0.033  (fraction) | (12) |
|  | Time to death | The time in days after which a patient-agent will die during the diagnostic pathway | Weibull(shape = 1.72, scale = 669.73, min = 0) (continuous) | Digitized KM-curve for patient subgroup chemotherapy with PD-L1 TPS score ≥ 50% (13) |
| **MTB** | Meeting schedule | The frequency that each MTB meet and discuss cases. | Weekly 3-hour meeting | (5), assumed to be identical across MTB-agents |
|  | Time required for each case | The time in minutes required to discuss one patient in the MTB. | Normal distribution(mean = 4, variance = 1.5, min = 0, max = 10) (continuous) | Expert opinion |
| **WGS facility** | Sequencing capacity | The number of biopsies that can be sequenced concurrently at any given moment | Unlimited | Assumption |
|  | WGS technical success rate | The proportion of biopsies were not successfully sequenced even though they contained enough tumor cells | Beta(p = 570, q = 28) (fraction) | (14) |
|  | Turnaround time WGS | Represents turnaround time of WGS in days | Truncated normal distribution (min = 7, max = 21, mean = 14, sigma = 3) (continuous) | Mean: (14)  Type of distribution, min, max, sigma is based on assumption. |
|  | Cost shallow WGS | The cost of conducting shallow WGS on one biopsy | 25% * Cost WGS (continuous) | Expert opinion |
|  | Shallow WGS quality assessment | The probability that a given biopsy fails the quality assessment by shallow WGS | Beta(p = 28, q = 570) (fraction) | (14) |
| **Hospital (academic, teaching, general)** | NGS technical success rate | Probability that an NGS panel is successfully conducted | Beta(p = 845.775, q = 49.225) (fraction) | (9) |
|  | Cost WGS | The cost of conducting WGS for one patient with NSCLC | 2925.25 euro (continuous) | (15) |
|  | Cost NGS panel | The cost of conducting one targeted gene panel for one patient with NSCLC. | The mean of reported costs for three different NGS panels  $cost=\frac{329.85 + 263.04 + 258.96}{3}=283.95$  (continuous) | (15) |
|  | Cost test ALK with IHC | The cost of conducting one IHC for an ALK rearrangement for one patient | 101.88 euro (continuous) | (15) |
|  | Cost test PD-L1 with IHC | The cost of conducting one IHC for the PD-L1 expression level for one patient | 93.74 euro (continuous) | (15) |
|  | Cost test EGFR Sanger Sequencing | The cost of conducting one Sanger Sequencing test for EGFR for one patient | 71.19 euro (continuous) | (15) |
|  | Cost test KRAS Sanger Sequencing | The cost of conducting one Sanger Sequencing test for KRAS for one patient | The mean of reported costs for two different platforms  $cost=\frac{71.19 + 63.47}{2}=67.33$  (continuous) | (15) |
|  | Turnaround time PD-L1 test general hospitals | The time in days from ordering PD-L1 test until receiving results in a general hospital for one patient | Discrete empirical distribution(min = 4, max = 10) (discrete) | Survey data, see section 1.1.3.2 and table 2. |
|  | Turnaround time PD-L1 test teaching hospitals | The time in days from ordering PD-L1 test until receiving results in a teaching hospital for one patient | Discrete empirical distribution(min = 5, max = 15) (discrete) | Survey data, see section 1.1.3.2 and table 2. |
|  | Turnaround time PD-L1 test academic hospitals | The time in days from ordering PD-L1 test until receiving results in an academic hospital for one patient | Discrete empirical distribution(min = 4, max = 15) (discrete) | Survey data, see section 1.1.3.2 and table 2. |
|  | Turnaround time molecular diagnostics general hospitals | The time in days from ordering molecular diagnostics until receiving results in a general hospital for one patient | Discrete empirical distribution(min = 10, max = 21) (discrete) | Survey data, see section 1.1.3.2 and table 2. |
|  | Turnaround time molecular diagnostics teaching hospitals | The time in days from ordering molecular diagnostics until receiving results in a teaching hospital for one patient | Discrete empirical distribution(min = 10, max = 23) (discrete) | Survey data, see section 1.1.3.2 and table 2. |
|  | Turnaround time molecular diagnostics academic hospitals | The time in days from ordering molecular diagnostics until receiving results in an academic hospital for one patient | Discrete empirical distribution(min = 10, max = 21) (discrete) | Survey data, see section 1.1.3.2 and table 2. |
|  | Treatment delay general hospitals | The time in days after concluding diagnostics and between treatment initiation in a general hospital for one patient | Discrete empirical distribution(min = 1 , max = 5) (discrete) | Survey data, see section 1.1.3.2 and table 2. |
|  | Treatment delay teaching hospitals | The time in days after concluding diagnostics and between treatment initiation in a teaching hospital for one patient | Discrete empirical distribution(min = 4, max = 9) (discrete) | Survey data, see section 1.1.3.2 and table 2. |
|  | Treatment delay academic hospitals | The time in days after concluding diagnostics and between treatment initiation in an academic hospital for one patient | Discrete empirical distribution(min = 0, max = 10) (discrete) | Survey data, see section 1.1.3.2 and table 2. |
|  | Sufficient Tumor cells in WGS biopsy | Probability that any of the biopsies taken for WGS contain enough tumor cells | Beta(p = 570, q = 297) (fraction) | (14) |
|  | Referral error | Probability that a patient for whom no biomarker was identified in the current hospital is not referred to either a teaching or academic hospital for further testing | 0 (fraction) | Assumption |

## Outcome stability


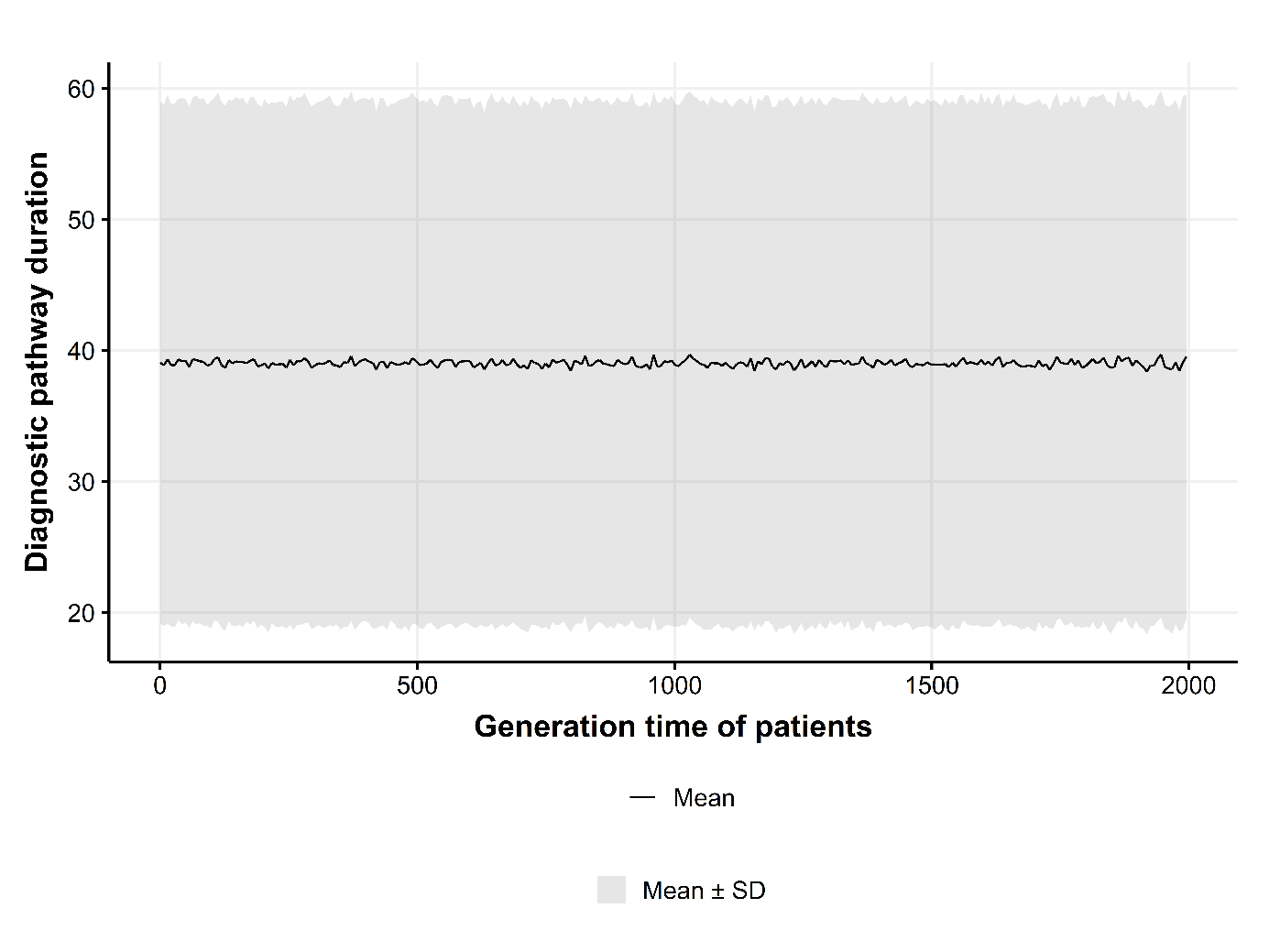


Figure 8. The duration of the diagnostic pathway for patients generated at different simulation times based on 500 simulation runs.

## Appendix bibliography

1. Ven M van de, IJzerman M, Retèl V, Harten W van, Koffijberg H. The nationwide implementation of Whole Genome Sequencing in oncoloy in the Netherlands [Internet]. AnyLogic Cloud. 2021 [cited 2021 Jan 26]. Available from: https://cloud.anylogic.com/model/6f5c67f2-1423-422a-be35-63f0f664cc77?mode=SETTINGS

2. Nederlandse Kankerregistratie [Netherlands Cancer Registry]. Incidentie, Niet-kleincellig longcarcinoom, 2017, Aantal [Incidence, Non-small cell lung cancer, 2017, Amount] [Internet]. Available from: https://www.iknl.nl/nkr-cijfers?fs%7Cepidemiologie_id=6&fs%7Ctumor_id=260&fs%7Cregio_id=161%2C159%2C157%2C164%2C158%2C160%2C165%2C163%2C167%2C166%2C156%2C162&fs%7Cperiode_id=106&fs%7Cgeslacht_id=15&fs%7Cleeftijdsgroep_id=67&fs%7Cjaren_na_diagnose_id=16&fs

3. National Comprehensive Cancer Network. Non-Small Cell Lung Cancer (version 6.2020) [Internet]. 2020. Available from: https://www.nccn.org/professionals/physician_gls/pdf/nscl.pdf

4. van de Ven M, Retèl VP, Koffijberg H, van Harten WH, IJzerman MJ. Variation in the time to treatment for stage III and IV non-small cell lung cancer patients for hospitals in the Netherlands. Lung Cancer. 2019;134(May):34–41.

5. PATH. Moleculaire tumor boards [Molecular tumor boards] [Internet]. [cited 2020 Jun 17]. Available from: https://www.netwerk-path.nl/index.php/tumor-boards

6. Kuijpers CCHJ, Hendriks LEL, Derks JL, Dingemans AMC, van Lindert ASR, van den Heuvel MM, et al. Association of molecular status and metastatic organs at diagnosis in patients with stage IV non-squamous non-small cell lung cancer. Lung Cancer. 2018 Jul 1;121:76–81.

7. Kerner GSMA, Schuuring E, Sietsma J, Hiltermann TJN, Pieterman RM, de Leede GPJ, et al. Common and Rare EGFR and KRAS Mutations in a Dutch Non-Small-Cell Lung Cancer Population and Their Clinical Outcome. Batra SK, editor. PLoS One [Internet]. 2013 Jul 29 [cited 2020 Sep 29];8(7):e70346. Available from: https://dx.plos.org/10.1371/journal.pone.0070346

8. Dietel M, Savelov N, Salanova R, Micke P, Bigras G, Hida T, et al. Real-world prevalence of programmed death ligand 1 expression in locally advanced or metastatic non–small-cell lung cancer: The global, multicenter EXPRESS study. Lung Cancer. 2019;134(February):174–9.

9. VanderLaan PA, Rangachari D, Majid A, Parikh MS, Gangadharan SP, Kent MS, et al. Tumor biomarker testing in non-small-cell lung cancer: A decade of change. Lung Cancer [Internet]. 2018;116(December 2017):90–5. Available from: https://doi.org/10.1016/j.lungcan.2018.01.002

10. Hirsch FR, Scagliotti G V., Mulshine JL, Kwon R, Curran WJ, Wu YL, et al. Lung cancer: current therapies and new targeted treatments. Lancet. 2017;389(10066):299–311.

11. Michels S, Scheel AH, Scheffler M, Schultheis AM, Gautschi O, Aebersold F, et al. Clinicopathological characteristics of RET rearranged lung cancer in European patients. J Thorac Oncol [Internet]. 2016 Jan 1 [cited 2020 Sep 29];11(1):122–7. Available from: http://dx.doi.org/10.1016/j.jtho.2015.09.016

12. Simmons CP, Koinis F, Fallon MT, Fearon KC, Bowden J, Solheim TS, et al. Prognosis in advanced lung cancer - A prospective study examining key clinicopathological factors. Lung Cancer [Internet]. 2015 [cited 2020 Jun 16];88(3):304–9. Available from: http://dx.doi.org/10.1016/j.lungcan.2015.03.020

13. Mok TSK, Wu YL, Kudaba I, Kowalski DM, Cho BC, Turna HZ, et al. Pembrolizumab versus chemotherapy for previously untreated, PD-L1-expressing, locally advanced or metastatic non-small-cell lung cancer (KEYNOTE-042): a randomised, open-label, controlled, phase 3 trial. Lancet. 2019;393(10183):1819–30.

14. Monkhorst K, Samsom K, Schipper L, Roepman P, Bosch L, Bruijn E de, et al. Validation of whole genome sequencing in routine clinical practice. ESMO Annu Meet [Internet]. 2020 [cited 2020 Sep 29];31:1189O. Available from: https://doi.org/10.1016/j.annonc.2020.08.083

15. Pasmans CTB, Tops BBJ, Steeghs EMP, Coupé VMH, Grünberg K, de Jong EK, et al. Micro-costing diagnostics in oncology: from single-gene testing to whole- genome sequencing. Expert Rev Pharmacoeconomics Outcomes Res [Internet]. 2021 May 6;21(3):413–4. Available from: https://doi.org/10.1080/14737167.2021.1917385
