# Supplementary material for: Developing a dynamic simulation model to support the nationwide implementation of whole genome sequencing in lung cancer
Source: BMC Med Res Methodol. 2022 Mar 27;22:83. doi: 10.1186/s12874-022-01571-3 (PMC8962015; doi:10.1186/s12874-022-01571-3)
Supplement: Supplementary file 2 — Additional file 2. The SIMULATE checklist [file 12874_2022_1571_MOESM2_ESM.docx]

# The SIMULATE checklist

An application of the SIMULATE checklist [1] accompanying the manuscript titled ‘Developing a dynamic simulation model to support the nationwide implementation of Whole Genome Sequencing in lung cancer‘ by van de Ven et al. (2021).

## System

The modelled system reflects a part of the healthcare system in the Netherlands. Specifically, the modelled system reflects the current diagnostic pathway for lung cancer patients. As the purpose of the model is to analyze the consequences of implementing Whole Genome Sequencing (WGS) at a national level, the modelled system includes multiple hospitals, as well as care providers involved with conducting WGS and providing clinical interpretations of the genetic information that WGS provides. In short, the stakeholders involved include patients, hospitals, the WGS facility, and Molecular Tumor Boards (MTB).

## Interaction

The model includes nonlinear and spatial relationship among its stakeholders. When patients are generated, they will select and move to a hospital in which they will undergo initial diagnostics. This hospital selection is based on distance. Moreover, hospitals may refer patients to other hospitals; whichever hospital they refer to is dependent on hospital type (academic, teaching, general) and distance. Similarly, hospitals send WGS reports corresponding to patients who received WGS to either one of the two nearest MTBs. These flows of agents lead to nonlinear behavior within the system and makes it difficult to predict the outcomes.

## Multilevel

The model takes a tactical and strategic perspective. While certain model components such as the modelled care processes are more of an operational nature, these are necessary to answer questions at the tactical and strategic level. Given that the model includes all hospitals that treat lung cancer patients in the Netherlands, it is able to provide valuable information both at the hospital level as well as at the national level. If so desired, the insights gained from the model can be interesting for individual hospital managers, but also policymakers at the national level.

## Understanding

A closed-form analytical approach would not able to address the research questions. This is primarily due to the randomness that is included in the model; patient characteristics, outcomes of diagnostics, time-to-events, costs, etc., all contain stochasticity. More traditional models such as Markov models or microsimulation models would not be able to reflect the multi-levelled nature of the system and research questions.

## Loops

The model contains feedback loops in two areas of the model: (1) feedback loops between hospitals (due to patient and referral flows), (2) feedback loops in diagnostic pathways within hospitals (e.g., if a test fails, it can be repeated). These feedback loops increase the nonlinear behavior of the system and may lead to queues within the modelled care processes. Ultimately, these queues can lead to delays and potentially prevent patients from receiving the care that was indicated for them.

## Agents

The model includes multiple agent populations: patients, hospitals, the WGS facility, MTBs, and WGS biopsies that are generated if a patient receives WGS. Beyond the hospital selection by patients, these agents are passive. In other words, the behavior of these agents is purely informed by relatively simple rules specified by the authors.

## Time

Time is a key component of the model. Delays caused by the modelled care processes influence the time-to-treatment, which is one of the primary endpoints for this study. Moreover, the patient population is an open population, meaning that patients are continuously being generated and added to the model.

## Emergence

While this study is not a full-fledged application of the model, the model has the potential to illuminate both the intended and unintended consequences of implementing WGS nationally in the healthcare system. For instance, by aiming to reflect a part of the real-world healthcare system, thereby including real-world constraints, it is able the consequences of these constraints and contrast them with the outcomes of unconstrained analyses, such as what is typically assumed in cost-effectiveness analyses.

# References

[1] D.A. Marshall, L. Burgos-Liz, M.J. Ijzerman, N.D. Osgood, W. V. Padula, M.K. Higashi, P.K. Wong, K.S. Pasupathy, W. Crown, Applying dynamic simulation modeling methods in health care delivery research - The SIMULATE checklist: Report of the ISPOR simulation modeling emerging good practices task force, Value Heal. 18 (2015) 5–16. https://doi.org/10.1016/j.jval.2014.12.001.
